# Social-biological influences on sleep duration among adult residents of Northeastern China

**DOI:** 10.1186/s12955-019-1111-3

**Published:** 2019-03-15

**Authors:** Yaxuan Ren, Yawen Liu, Tingyu Meng, Wenshu Liu, Yichun Qiao, Yulu Gu, Yong Li, Yunkai Liu, Yaqin Yu, Yi Cheng

**Affiliations:** 1 Department of Epidemiology and Biostatistics, School of Public Health of Jilin University, Changchun, 130021 China; 2 Cardiovascular Center, The First Hospital of Jilin University, Changchun, 130021 China

**Keywords:** Sleep duration, Adults, Influencing factors, China

## Abstract

**Background:**

Cold climates traditionally have conferred long sleep duration in the residents in northeast China; however, modern lifestyle reduces sleep duration. In this study, we investigated social-biological factors influencing sleep duration in the adult residents in northeast China.

**Methods:**

This study was performed using data from the Investigation of Chronic Disease Morbidity Rate and Risk Factors of Adults in Jilin Province, China. Associations between sleep duration and indices of demographic characteristics, health-related behaviors, and disease history in adult residents were analyzed using univariate analysis and multivariate logistic regression analysis.

**Results:**

The mean sleep duration was 7.24 h. Of the 21,435 participants, approximately 53.4% had short sleep duration (sleep duration per day < 7 h), and 10.5% had long sleep duration (sleep duration per day > 9 h). There were associations between short sleep duration and indices, including age, place of residence, marital status, educational level, alcohol drinking, dietary, obesity, and history of coronary heart disease (CHD) or myocardial infarction (MI). There existed associations of long sleep duration with indices, such as age, place of residence, occupation, educational level, average monthly earnings, and physical exercise.

**Conclusion:**

Short sleep duration is common among residents in northeast China. Age, place of residence, and educational level are implicated in both short sleep duration and long sleep duration. Short sleep duration inclines to link with the indices (marital status, alcohol drinking, dietary, obesity, and history of CHD or MI). However, long sleep duration is relevant to the indices (occupation, average monthly earnings, and physical exercise).

## Background

Sleep maintains human health by regulating physiological and psychological functions [1]. Sleep disorders have been becoming increasingly common worldwide [2], afflicting 56% of Americans, 31% of western Europeans, 23% of Japanese, and 42% of Chinese [3]. Sleep disorders are a group of conditions that affect the ability to sleep well on a regular basis [4]. General symptoms of sleep disorders include difficulty falling or staying asleep, oversleeping, daytime fatigue, strong urge to take naps during the day, irritability or anxiety, lack of concentration, or depression [5].

Insufficient sleep syndrome and hypersomnia are still the main sleep disorders [6], despite advances in therapy that has been achieved. Abnormal sleep duration constitutes the same factor between insufficient sleep syndrome and hypersomnia [7]. Insufficient sleep syndrome is the most common cause of daytime sleepiness among the general population [8]. Persons with insufficient sleep syndrome routinely spend less than 8 h in bed at night, and have their symptoms ameliorated if they sleep for a longer period of time [9]. This syndrome results from insufficient duration of sleep nightly, affecting all systems of the body and giving rise to cognitive impairment [10]. In contrast, people with hypersomnia might require as many as 10 to 12 h of sleep per night to feel their best [11]. For people with hypersomnia, oversleeping may cause anxiety, back pain, headache, and memory problems [12]. Moreover, abnormal sleep duration correlates with negative health outcomes: short sleep duration is associated with diseases, such as hypertension [13], diabetes [14], or dyslipidemia [15]; and long sleep duration is associated with diseases, including hypertension [16], diabetes [17], obesity [18], metabolic syndrome [19], or dyslipidemia [20]. Thus, apart from medications, finding sleep-duration-influencing factors is necessary for controlling sleep disorders.

Investigations on sleep duration in China have been focusing on specific populations (children, students, elderly, and night workers) [21–24], rather than focusing on general population. Cold climates traditionally have conferred long sleep duration in the residents in northeast China [25]. However, the accelerating pace of modernization and increasing social pressure have interfered the sleep duration of general population in China. As such, the purpose of the current study was to investigate social-biological factors related to sleep duration among adult in northeast China.

## Methods

### Study population

A multistage stratified cluster random sampling method was used to select our study population from data collected from the project (Investigation of Chronic Disease Morbidity Rate and Risk Factors of Adults in Jilin Province) that we conducted in 2012. We randomly selected 32 districts/counties in nine cities/prefectures of Jilin Province. A total of 22,600 permanent residents aged 18–79 years, who have lived in Jilin Province for more than 6 months, were included. After incomplete or incorrect information was removed, a total of 21,435 participants were remained in our final analysis. The questionnaire was designed by the faculty in the Department of Epidemiology and Biostatistics, School of Public Health, Jilin University. All participants provided written informed consent prior to participating in the survey. This study was approved by the ethics committee of Jilin Center for Disease Control and Prevention.

### Measurements

The content relative to sleep duration in questionnaire included the following question: “how many hours do you spend sleeping on an average day?”. Sleep durations were collected from the answers. Average sleep duration was classified as < 7 h of sleep (short sleep duration), 7~9 h of sleep (sufficient sleep), and > 9 h of sleep (long sleep duration) [26].

The questionnaire also included the demographic characteristics of the respondents (sex, age, ethnicity, place of residence, marital status, educational level, occupation, and average monthly earnings), health related behaviors (cigarette smoking, alcohol drinking, dietary, and physical exercise), and disease history (hypertension, diabetes, hyperlipidemia, and coronary heart disease [CHD] or myocardial infarction [MI]). Age was recoded into three categories: 18~44 years old (young), 45~59 years old (middle age), and 60~79 years old (elderly).

Anthropometric measurements, including height, weight, and blood pressure, were performed by trained staffs. The body mass index (BMI) was calculated by dividing the weight by the square of the height. Based on recommendations from the Working Group on Obesity in China [27], BMI was categorized as follows: < 18.5: underweight, 18.5~23.9: normal, 24.0~27.9: overweight, and ≥ 28.0: obesity.

All investigators and laboratory technicians had been uniformly trained. A pilot survey was conducted prior to the formal investigation, and necessary amendments were made. Sphygmomanometers, weighing scales, and glucose meters were calibrated before they were used. After questionnaires were collected during the formal investigation, quality control persons reviewed those questionnaires on site and immediately corrected errors, such as wrong filling, leaked filling, and unclear handwriting. If the errors were not able to be corrected on site, error-existing questionnaires were discarded. Data were entered using double cross input, and the third inspection of the data was performed before analyses of data.

### Statistical analysis

The database was established by Epidata3.1, and all statistical analysis were performed using SPSS 19.0. Values of sleep duration were reported as mean ± *SD*. Mean and standard deviation had been adjusted by a complex weighed computation. For comparisons of indices, analysis of variance was used in quantitative data, and the chi-square test was used in categorical data. Univariate analysis was used to evaluate sleep-duration-influencing factors. Multivariate logistic regression was used to assess the associations between factors and sleep duration. The odds ratio (*OR*) and 95% confidence interval (*CI*) were calculated. *P* < 0.05 indicated statistical significance.

## Results

A total of 21,435 participants were enrolled in this investigation. Among the participants, participants with Han nationality represented 92.7%, and married/cohabitated participants accounted for 85.4%. The mean of sleep duration per day was 7.24 h in the total participants. Differences of sleep duration significantly existed in indices, including age, ethnicity, place of residence, marital status, educational level, occupation, and average monthly earnings. There was no significant difference between male and female in sleep duration (Table 1). Among all the participants, 11,436 participants (53.4%) had short sleep duration, and 2260 participants (10.5%) had long sleep duration.

**Table 1:** Sleep duration among different population groups

Characteristics	N (%)	Sleep duration(h) $\overline{x}$*± s*	*F*	*P*
Sex				
Male	10,337 (48.2)	7.26±1.491	1.83	0.067
Female	11,098 (51.8)	7.22±1.597		
Age				
Young	9043 (42.2)	7.50±1.331	353.95	< 0.001
Middle age	8277 (38.6)	6.90±1.557		
Elderly	4115 (19.2)	6.86±1.777		
Ethnicity				
Han	19,865 (92.7)	7.25±1.543	3.47	0.001
Others	1570 (7.3)	7.10±1.458		
Place of residence				
Urbanite	11,152 (52.0)	7.14±1.447	9.21	< 0.001
Countryside	10,283 (48.0)	7.37±1.623		
Marital status				
Marriage /Cohabitation	18,316 (85.4)	7.22±1.524	65.44	< 0.001
Unmarried	1693 (7.9)	7.57±1.344		
Divorce/Divorcee/Separated spouse	388 (1.8)	6.83±1.589		
Widow/ Widower	1038 (4.8)	6.63±1.854		
Occupation				
Manual worker	12,046 (56.2)	7.30±1.560	22.19	< 0.001
Mental worker	4211 (19.6)	7.27±1.246		
Others	5178 (24.2)	7.01±1.672		
Educational level				
Primary school or below	6236 (29.1)	7.11±1.816	19.89	< 0.001
Junior middle school	6125 (28.6)	7.32±1.488		
Senior middle school	5559 (25.9)	7.18±1.433		
Undergraduate or above	3515 (16.4)	7.34±1.190		
Average monthly income (¥)				
< 500	4304 (20.1)	7.18±1.726	7.55	0.001
500-	14,149 (66.0)	7.22±1.610		
3000-	2140 (10.0)	7.35±1.464		

Mean and standard deviation has been adjusted by complex weighed computation in sleep duration

We based on the 17 independent variables that were listed in questionnaire to investigate short-sleep-duration-influencing factors using univariate analysis. We found that short sleep duration was associated with the variables (age, place of residence, marital status, occupation, educational level, average monthly earnings, cigarette smoking, alcohol drinking, dietary, physical exercise, BMI, history of hypertension, diabetes, and CHD or MI) (all *P* < 0.05) (Table 2). Univariate analysis further revealed that there were increased risks of short sleep duration in the participants with factors, such as urban (*OR* = 1.12, 95%*CI* = 1.04–1.20), divorced/ separated (*OR* = 1.75, 95%*CI* = 1.39–2.19), widowed (*OR* = 2.18, 95%*CI* = 1.87–2.50), manual (*OR* = 1.26, 95%*CI* = 1.15–1.38), cigarette smoking (*OR* = 1.30, 95%*CI* = 1.20–1.40), alcohol drinking (*OR* = 1.11, 95%*CI* = 1.03–1.20), history of hypertension (*OR* = 1.60, 95%*CI* = 1.48–1.72), diabetes (*OR* = 1.41, 95%*CI* = 1.29–1.53), and CHD or MI (OR = 1.68, 95%CI = 1.49–1.90). Moreover, short sleep duration was more serious in participants with these factors (middle-age: *OR* = 2.27, 95%*CI* = 2.10–2.44; elderly: *OR* = 2.58, 95%*CI* = 2.33–2.85; overweight: *OR* = 1.21, 95%*CI* = 1.11–1.30; and obesity: *OR* = 1.34, 95%*CI* = 1.21–1.49). In comparison, protective factors for short sleep duration were unmarried (*OR* = 0.59, 95%*CI* = 0.51–0.68), regular diet (*OR* = 0.78, 95%*CI* = 0.71–0.85), proper physical exercise (*OR* = 0.70, 95%*CI* = 0.64–0.77), junior middle school level (*OR* = 0.68, 95%*CI* = 0.62–0.75), senior middle school level (*OR* = 0.73, 95%*CI* = 0.67–0.81), undergraduate or above level (*OR* = 0.48, 95%*CI* = 0.43–0.54), and average monthly earnings of ¥500~ (*OR* = 0.77, 95%*CI* = 0.70–0.84) and ¥3000~ (*OR* = 0.65, 95%*CI* = 0.57–0.74) (Table 2).

**Table 2:** Univariate factor analysis of short sleep duration and long sleep duration

Factors	Short sleep duration				Long sleep duration			
	*χ* ^*2*^		*OR*	(95%*CI*)	*χ* ^*2*^		*OR*	(95%*CI*)
Sex	2.	18			5.	93*		
Male			1.05	(0.98, 1.13)			0.87	(0.78, 0.97)
Female			1				1	
Age	566.	10*			17.	57*		
Young			1				1	
Middle age			2.27	(2.10, 2.44)			0.86	(0.77, 0.97)
Elderly			2.58	(2.33, 2.85)			1.17	(1.01, 1.34)
Ethnicity	2.	44			6.	92*		
Han			0.90	(0.79, 1.03)			1.33	(1.08, 1.64)
Others			1				1	
Place of residence	9.	59*			66.	07*		
Urbanite			1.12	(1.04, 1.20)			0.63	(0.56, 0.70)
Countryside			1				1	
Marital status	187.	08*			2.	72		
Marriage /Cohabitation			1				1	
Unmarried			0.59	(0.51, 0.68)			1.12	(0.93, 1.34)
Divorce/Divorcee/Separated spouse			1.75	(1.39, 2.19)			0.88	(0.56, 1.37)
Widow/ Widower			2.18	(1.87, 2.50)			1.14	(0.89, 1.45)
Occupation	87.	80*			52.	45*		
Mental worker			1				1	
Manual worker			1.26	(1.15, 1.38)			1.70	(1.46, 1.98)
Others			1.67	(1.50, 1.85)			1.83	(1.52, 2.21)
Educational level	180.	35*			125.	52*		
Primary school or below			1				1	
Junior middle school			0.68	(0.62, 0.75)			0.74	(0.64, 0.85)
Senior middle school			0.73	(0.67, 0.81)			0.52	(0.45, 0.61)
Undergraduate or above			0.48	(0.43, 0.54)			0.43	(0.36, 0.51)
Average monthly income (¥)	51.	70*			53.	30*		
< 500			1				1	
500-			0.77	(0.70, 0.84)			0.61	(0.53, 0.70)
3000-			0.65	(0.57, 0.74)			0.60	(0.49, 0.75)
Cigarette smoking	63.	29*			0.	89		
Never			1				1	
Once			1.44	(1.28, 1.63)			1.07	(0.89, 1.30)
Currently			1.30	(1.20, 1.40)			0.97	(0.86, 1.10)
Alcohol drinking	7.	81*			13.	04*		
No			1				1	
Yes			1.11	(1.03, 1.20)			0.80	(0.71, 0.90)
Dietary	30.	58*			3.	90*		
Irregular			1				1	
Regular			0.78	(0.71, 0.85)			0.87	(0.75, 0.99)
Physical exercise	95.	45*			40.	13*		
Never			1				1	
Sometimes			0.70	(0.64, 0.77)			0.70	(0.61, 0.81)
Often			1.12	(1.03, 1.21)			0.70	(0.61, 0.79)
BMI	68.	11*			11.	94*		
Normal			1				1	
Underweight			0.66	(0.54, 0.81)			1.20	(1.08, 1.33)
Overweight			1.21	(1.11, 1.30)			0.85	(0.75, 0.96)
Obesity			1.34	(1.21, 1.49)			1.02	(1.01, 1.03)
Hypertension	147.	91*			1.	45		
No			1				1	
Yes			1.60	(1.48, 1.72)			1.08	(0.96, 1.21)
Diabetes	58.	15*			0.	001		
No			1				1	
Yes			1.41	(1.29, 1.53)			1.00	(0.86, 1.16)
Hyperlipidemia	1.	01			3.	33		
No			1				1	
Yes			1.04	(0.96, 1.23)			1.13	(0.99, 1.29)
CHD or MI^a^	69.	96*			7.	63*		
No			1				1	
Yes			1.68	(1.49, 1.90)			1.30	(1.08,1.57)

*:*P*<0.05

a:*CHD* Coronary Heart Disease, *MI* Myocardial Infarction

1:the reference cell

For long sleep duration, univariate analysis showed factors, such as sex, age, ethnicity, place of residence, occupation, educational level, average monthly earnings, alcohol drinking, dietary, physical exercise, BMI, and history of CHD or MI, were associated with long sleep duration (all *P* < 0.05) (Table 2). The increased risk factors of long sleep duration were consisted of such variables (elderly: *OR* = 1.17, 95%*CI* = 1.01–1.34; Han nationality: *OR* = 1.33, 95%*CI* = 1.08–1.64; manual: *OR* = 1.70, 95%*CI* = 1.46–1.98; underweight: *OR* = 1.20, 95%*CI* = 1.08–1.33; obesity: *OR* = 1.02, 95%*CI* = 1.01–1.03; and history of CHD or MI: *OR* = 1.30, 95%*CI* = 1.08–1.57). Moreover, the decreased risk factors of long sleep duration were composed of the variables (male: *OR* = 0.87, 95%*CI* = 0.78–0.97; middle age: *OR* = 0.86, 95%*CI* = 0.77–0.97; urban: *OR* = 0.63, 95%*CI* = 0.56–0.70; junior middle school level: *OR* = 0.74, 95%*CI* = 0.64–0.85; senior middle school level: *OR* = 0.52, 95%*CI* = 0.45–0.61; undergraduate or above level: *OR* = 0.43, 95%*CI* = 0.36–0.51; alcohol drinking: *OR* = 0.80, 95%*CI* = 0.71–0.90; regular diet: *OR* = 0.87, 95%*CI* = 0.75–0.99; sometimes physical exercise: *OR* = 0.70, 95%*CI* = 0.61–0.81; often physical exercise: *OR* = 0.70, 95%*CI* = 0.61–0.79; overweight: *OR* = 0.85, 95%*CI* = 0.75–0.96; and average monthly earnings of ¥500~: *OR* = 0.61, 95%*CI* = 0.53–0.70, and ¥3000~: *OR* = 0.60, 95%*CI* = 0.49–0.75) (Table 2).

We further used multivariate logistic regression analysis to validate sleep-duration-influencing factors. We identified that the increased risks of short sleep duration were associated with the factors (divorced/separated: *OR* = 1.381, 95%*CI* 1.193–2.272; widowed: *OR* = 1.433, 95%*CI* = 1.181–1.738; alcohol drinking: *OR* = 1.198, 95%*CI* = 1.066–1.347; history of CHD or MI: *OR* = 1.269, 95%*CI* = 1.114–1.446; middle-aged: *OR* = 2.008, 95%*CI* = 1.778–2.268; elderly: *OR* = 2.066, 95%*CI* = 1.751–2.438; and obesity: *OR* = 1.192, 95%*CI* = 1.035–1.373). However, the protective factors for short sleep duration consisted of the variables (urban: *OR* = 0.782, 95%*CI* = 0.698–0.876; regular diet: *OR* = 0.741, 95%*CI* = 0.650–0.846; junior middle school level: *OR* = 0.854, 95%*CI* 0.747–0.976; and undergraduate or above level: *OR* = 0.777, 95%*CI* = 0.630–0.959) (Table 3). In addition, we found that the increased risk factors of long sleep duration were composed of urban (*OR* = 1.386, 95%*CI* 1.131–1.697) and others occupation (*OR* = 1.713, 95%*CI* 1.227–2.392). Conversely, the protective factors for long sleep duration were composed of such variables (middle age: *OR* = 0.806, 95%*CI* = 0.674–0.964; senior middle school level: *OR* = 0.642, 95%*CI* = 0.499–0.826; undergraduate or above level: *OR* = 0.607, 95%*CI* = 0.416–0.885; average monthly earnings of ¥500~2999: *OR* = 0.800, 95%*CI* = 0.658–0.971; often physical exercise: *OR* = 0.741, 95%*CI* = 0.616–0.893; and overweight: *OR* = 0.791, 95%*CI* = 0.664–0.942) (Table 4).

**Table 3:** Multivariate logistic regression analysis of factors associated with short sleep duration

Factors	*β*		*χ* ^*2*^		*P*		*OR*		95% *CI*
Age									
Young	0		128.	686	< 0.	001	1		
Middle age	0.	697	125.	842	< 0.	001	2.	008	(1.778, 2.268)
Elderly	0.	726	73.	741	< 0.	001	2.	066	(1.751, 2.438)
Place of residence									
Urbanite	−0.	246	18.	077	< 0.	001	0.	782	(0.698, 0.876)
Countryside	0						1		
Marital status									
Marriage /Cohabitation	0		21.	998	< 0.	001	1		
Unmarried	0.	139	0.	059	0.	807	1.	149	(0.771, 1.396)
Divorce/Divorcee/Separated spouse	0.	323	13.	340	< 0.	001	1.	381	(1.193, 2.272)
Widow/ Widower	0.	360	9.	209	0.	002	1.	433	(1.181, 1.738)
Educational level									
Primary school or below	0		7.	865	0.	049	1		
Junior middle school	−0.	158	5.	368	0.	021	0.	854	(0.747, 0.976)
Senior middle school	−0.	118	2.	419	0.	120	0.	889	(0.766, 1.031)
Undergraduate or above	−0.	252	5.	537	0.	019	0.	777	(0.630, 0.959)
Drinking									
No	0						1		
Yes	0.	181	9.	229	0.	002	1.	198	(1.066, 1.347)
Dietary									
Irregular	0						1		
Regular	−0.	300	19.	799	< 0.	001	0.	741	(0.650, 0.846)
BMI									
Normal	0		9.	046	0.	029	1		
Underweight	−0.	134	0.	844	0.	358	0.	875	(0.659, 1.163)
Overweight	−0.	021	0.	149	0.	700	0.	979	(0.877, 1.092)
Obesity	0.	176	5.	932	0.	015	1.	192	(1.035, 1.373)
CHD or MI^a^									
No	0						1		
Yes	0.	238	12.	765	< 0.	001	1.	269	(1.114, 1.446)

^a^ *CHD* Coronary Heart Disease, *MI* Myocardial Infarction

1:the reference cell

**Table 4:** Multivariate logistic regression analysis of factors associated with long sleep duration

Factors	*β*		*χ* ^*2*^		*P*		*OR*		95% *CI*
Sex									
Male	−0.	176	3.	595	0.	058	0.	839	(0.700, 1.006)
Female	0						1		
Age									
Young	0		5.	853	0.	054	1		
Middle age	−0.	216	5.	595	0.	018	0.	806	(0.674, 0.964)
Elderly	−0.	127	1.	081	0.	299	0.	881	(0.694, 1.119)
Ethnicity									
Han	0.	272	3.	177	0.	075	1.	312	(0.973, 1.769)
Others	0						1		
Place of residence									
Urbanite	0.	326	9.	952	0.	002	1.	386	(1.131, 1.697)
Countryside	0						1		
Occupation									
Mental worker	0		13.	321	0.	001	1		
Manual worker	0.	160	1.	264	0.	261	1.	174	(0.888, 1.552)
Others	0.	538	10.	017	0.	002	1.	713	(1.227, 2.392)
Educational level									
Primary school or below	0		12.	837	0.	005	1		
Junior middle school	−0.	190	3.	230	0.	072	0.	827	(0.672, 1.017)
Senior middle school	−0.	443	11.	895	0.	001	0.	642	(0.499, 0.826)
Undergraduate or above	−0.	450	6.	741	0.	009	0.	607	(0.416, 0.885)
Average monthly earnings (¥)									
< 500	0		5.	201	0.	074	1		
500-	−0.	223	5.	093	0.	024	0.	800	(0.658, 0.971)
3000-	−0.	153	0.	583	0.	445	0.	858	(0.579, 1.271)
Physical exercise									
Never	0		11.	150	0.	004	1		
Sometimes	−0.	025	0.	051	0.	821	0.	975	(0.782, 1.215)
Often	−0.	300	9.	991	0.	002	0.	741	(0.616, 0.893)
BMI									
Normal	0		8.	822	0.	032	1		
Underweight	0.	129	0.	358	0.	550	1.	138	(0.745, 1.737)
Overweight	−0.	234	6.	927	0.	008	0.	791	(0.664, 0.942)
Obesity	−0.	034	0.	098	0.	754	0.	967	(0.781, 1.196)

1:the reference cell

## Discussion

To the best of our knowledge, this is the first study to investigate the relationship between sleep duration and the indices of demographic characteristics, health-related behaviors, and disease history in adult residents in northeast China. We found that the mean of sleep duration per day (7.24 h) in Jilin province was less than that in China in 2002 (8 .3h) [28] and 2007 (8 .1h) [29]; moreover, the rate of short sleep duration (sleep duration per day < 7 h) was 53.4%, and the rate of long sleep duration (sleep duration per day > 9 h) was 10.5%. Compared with long sleep duration, short sleep duration is common in adult residents in northeast China.

Negative associations of short sleep duration with factors (education level, family income, and leisure-time physical activity) and positive associations with factors (number of live births, history of night shift work, and certain chronic diseases) have been found in Shanghai, China [30]. Our results supported their conclusion other than live births and history of night shift work. Sun W. et al. [31] reveal short sleep duration is associated with increasing obesity in men, and long sleep duration is associated with obesity in women in Beijing; however, we did not find the difference between man and woman with respect to increasing obesity in Jilin province. Previous studies show rural residents have longer sleep duration than urban residents in Tianjin [32], Shanghai [30], and Beijing [33], China, but our results showed urban residents slept longer than those who lived in rural, partially because Jilin is an agricultural province, and rural residents have to spend more time in labor [34]. Similar to the discoveries of Troxel WM et al. [35], our results also showed divorce/divorcee/separation or widow/widower were susceptible to have short sleep duration. In accordance with the discovers of Foti KE et al. [36], we also found alcohol drinking and irregular diet were risk factors for short sleep duration.

Long sleep duration is negatively associated with age and education level [30]. Our study further found middle-age, senior middle school level, and undergraduate or above level were the protective factors for long sleep duration. Previous studies have found that physical activity is associated with long sleep duration [37], and in our study, people who never performed physical activity slept longer than people who performed physical activity frequently. However, Ferranti R et al. [38] and Foti KE et al. [36] indicate that sleep duration is not associated with physical activity. Thus, the relationship between sleep duration and physical activity remains elusive. Similar to the discovery of Sun W et al. [31] and Nagai et al. [39], our results supported no association between long sleep duration and obesity. Additionally, rural residents were more likely to have long sleep duration than urban residents in Guizhou province, China [40]. However, urban residents slept longer than rural residents in Jilin province.

Our study provides some inspirations for the sleep policy makers. In the future intervention work, factors (such as physical exercise) that may affect sleep duration should be prevented and corrected for people with different characteristics to improve sleep quality. At the same time, the government should increase the education of the impact of sleep on health, and advocate a healthy and adequate sleep.

There are some limitations in our study. First, the data in our study were based on self-reported questionnaires; therefore, the accuracy of the reported results cannot be determined. Second, there may be errors in anthropometric measurements, although the large sample size and quality control could partly compensate for the errors. Third, the temporality of associations cannot be determined because of the cross-sectional nature of this study design.

## Conclusions

Short sleep duration is common among residents in northeast China. Age, place of residence, and educational level are implicated in both short sleep duration and long sleep duration. Short sleep duration inclines to link with the indices (marital status, alcohol drinking, dietary, obesity, and history of CHD or MI). However, long sleep duration is relevant to the indices (occupation, average monthly earnings, and physical exercise).

## Abbreviations

BMI: Body mass index
CHD: Coronary heart disease
CI: Confidence interval
MI: Myocardial infarction
OR: Odds ratio
SD: Standard deviation
